# Reprising the *globalization* dimensions of international health

**DOI:** 10.1186/s12992-018-0368-3

**Published:** 2018-05-18

**Authors:** Ronald Labonté

**Affiliations:** School of Epidemiology and Public Health, University of Ottawa, 600 Peter Morand Crescent, Ottawa, K1G 5Z3 Canada

**Keywords:** Globalization, International health, Global health, Neoliberalism

## Abstract

Globalization is a fairly recent addition to the panoply of concepts describing the internationalization of health concerns. What distinguishes it from ‘international health’ or its newer morphing into ‘global health’ is a specific analytical concern with how globalization processes, past or present, but particularly since the start of our neoliberal era post-1980, is affecting health outcomes. Globalization processes influence health through multiple social pathways: from health systems and financing reforms to migration flows and internal displacement; via trade and investment treaties, labour market ‘flexibilization’, and the spread of unhealthy commodities; or through deploying human rights and environment protection treaties, and strengthening health diplomacy efforts, to create more equitable and sustainable global health outcomes. *Globalization and Health* was a pioneer in its focus on these critical facets of our health, well-being, and, indeed, planetary survival. In this editorial, the journal announces a re-focusing on this primary aim, announcing a number of new topic Sections and an expanded editorial capacity to ensure that submissions are ‘on target’ and processed rapidly, and that the journal continues to be on the leading edge of some of the most contentious and difficult health challenges confronting us.

*Globalization and Health* launched in 2005 with a focus on research, policy studies, and commentaries exploring how globalization processes affect health through their impact on health systems and, importantly, through affecting social, economic, commercial, and political determinants of health. I was quick to become a regular contributor (starting in 2007), worked for years as a reviewer, stepped onto the Editorial Board in 2012, served as Deputy Editor a few years later, and now, with Greg Martin, as one of the Editors-in-Chief.

Globalization is one of those messy and contested social constructs that only emerged with normative force in the 1980s. PubMed archives does cite a single reference to the term dating back to 1925, but annual ‘globalization’ key words did not start to swell until the late 1980s. The number peaked in 2015 at almost 6000 before a rather sharp downwards fall in 2017, likely a response to what some argue is a post-Trump collapse in globalization (I contend it is more a tarnishing in the term’s coinage than in its actual practices). In anodyne terms, globalization describes the multiple processes by which nations, businesses and people are increasingly connected and interdependent across the globe, through increased economic integration and communication exchange, cultural diffusion, and travel. Significantly, perhaps, the term was first popularized in the 1980s to describe global market integration, hinting that economic practices were the driving force behind contemporary globalization [1]. ‘Contemporary’ is an important adjective, since our past 40 years of what some have described as ‘hyperglobalization’ [2] and others as ‘neoliberal globalization’ [3] is not the first time in human history that people have traded, migrated, exchanged, colonized, or exploited across borders [4]. But there are differences: We are now experiencing at a global scale a “world shrinkage, of distances getting shorter, things moving closer” [5], with communicative technologies connecting peoples as never before and time appearing to accelerate at ever greater rates. As a paper in this journal a few years ago expressed, we might approach these integrating changes as a ‘globalist’ (all is good) or ‘skeptic’ (all is bad, or at least not so good), but inevitably we are all challenged to be ‘transformationalists’, accepting that globalization processes can be both good and bad but are irrevocably changing how we lead our lives and understand our place in the world [6].

As with globalization writ large so, too, with globalization and health as one of its important subscripts, and one that PubMed finds is still experiencing an uptick. Historians recount how health and disease have long been fellow travelers along the migratory pathways of people, goods, and capital. Similarly, and for well over the past century, there have been efforts to address health risks faced by some of the world’s most vulnerable populations, driven by amalgams of humanitarian and communitarian ethos, often with large doses of commercial and mercantilist interests [7]. For much of the last century, ‘international health’ – a concern with high disease burdens in other, generally poorer, countries – dominated much of our ‘global’ public health research and practice. It still does, as indeed it should, given the persisting inequities in health status and resources for health within and between countries. But health experiences that manifest within borders have causes and consequences that are inherently global in nature [8], a theme that should resonate with both readers of, and contributors to, this journal. But as I step into a new role as one of the Editors-in-Chief, and after lengthy discussions with several key editorial members, it is timely to reassert the journal’s unique niche and mandate within a burgeoning global health disciplinary field.

## Scoping the domains of journal interest

Stated most simply, submissions to the journal need to attend to the *globalization* aspect of what they are reporting on, and not simply address an *international* health topic. In truth, over the 13 years of submissions and acceptances, many articles have been more international in content, dealing with health systems or disease concerns within low- or middle-income countries. Papers such as these will still be invited but with a *caveat* that applies to all new submissions to the journal: All submissions must, in some fashion, address inherently global features of the topic they are addressing, including how globalization processes affect the research or policy issues the paper is interrogating. Single country studies are still invited, but only if such studies clearly state how the topic being explored is shaped by global/globalization mechanisms, and/or how findings may generalize or transfer to other countries or have relevance to global level policy discourse.

As in the past, we encourage all contributions to adopt, whenever possible, an intersectional approach in their analyses and/or commentaries, to allow for a better understanding of the multiple dimensions of social stratification (e.g. gender, racialization, socioeconomic status, class/caste, geographic location) that are affected by globalization processes, in turn differentially affecting health outcomes.

To further assist contributors in determining if there is a good fit between what they wish to submit and the journal’s slightly tightened mandate, journal editors have spent some time revising and adding to the existing Sections in which we are actively soliciting contributions, which we describe below.

### Development

Development theories and development assistance have been fixtures on the global health landscape for decades. Although health was originally given low development priority, its rise in the Millennium Development Goals and position in the new Sustainable Development Goals (SDGs) draws attention to the many controversies surrounding health development assistance, including the complex forms it takes (vertical, diagonal, horizontal), the lack of consistency over time, disbursements driven by donor interests rather than need, high transaction costs of poor donor coordination, a ‘charity’ rather than ‘entitlement’ approach, the rise of global philanthropies, and the lack of coherence between donors’ aid and their international trade/macroeconomic policies. In recent years, the role of micro-financing has been advanced as a key development strategy, although it remains controversial; and ‘social impact investing’ (where private speculators invest for global social/public good with the expectation of profit) is inserting a market logic into previously humanitarian notions of assistance or obligation. Papers submitted under this topic will focus on all forms and underlying theories of financial transfers from richer to poorer nations, and how these affect health outcomes, health systems and progress towards the SDH goals and targets.

### Disease

Diseases increasingly have cross-border causes and consequences. Global health security has become a foreign policy issue for many countries, linked to risks of pandemic influenza, and XDR (extreme drug resistant) and AMR (antimicrobial resistant) infections; and affected by weak health systems, population movements and lack of access to medicines. Noncommunicable diseases (NCDs) have recently become foci for international cooperation, policy/program development and knowledge sharing. ‘Vectors’ (social determinants) for NCDs include such globalization-related pathways as trade (and trade treaties), foreign investment (and investment treaties), and economic growth and urbanization associated with global economic integration. Most papers submitted to this journal are presumed to utilize some health data; papers submitted under this topic will have either (one or more) infectious disease or NCD as their particular focus, applying epidemiological or other research methods to examine the magnitude, distribution and globalization-related risk factors affecting their prevalence, severity and/or population distribution.

### Economics and trade

The increasingly interconnected and interdependent global economy as well as the changing nature of trade across borders impact development (both positively and negatively) with important implications for health the world over. Economic policies for the past four decades have largely embodied neoliberal theories that are subject to increasing empirical and theoretical scrutiny, with widely accepted concerns over their impact on inequality and poverty. Economic integration and trade and investment liberalization are defining features of contemporary globalization, and have given rise to new global supply chains, creating both health opportunities and risks. As with neoliberal economics, how trade and investment treaties impact on health outcomes within and between countries is hotly debated. A related outcome of trade and global market integration is the increased size and power of transnational corporations, where a few often dominate in different economic sectors, from food and drinks products, to banking and finance, to extractive industries, to health technologies including pharmaceuticals. Papers submitted under this topic explore these topics, and provide research, commentary and discussion needed to inform future health-equity enhancing macroeconomic policies and trade and investment rules; and the related power and influence on health exerted by multinational and transnational corporations.

### Environment

Ecosystems often cross borders, and their ecologies are heavily impacted by globalizing processes: climate change, resource depletion, pollution or, more positively, sustainable agriculture or renewable energy initiatives. Globalization plays a role (for better and for worse) in the health or vitality of ecosystems, which in turn plays a role in human health with feedback loops creating complex pathways of causality. Sustainable development has become the dominant theme of the post-2015 development agenda, as the potential of broad-based ecological crises keeps global environmental issues high on the global policy agenda. Papers submitted under this topic examine pathways by which globalization processes (e.g. trade, investment, economic growth, population growth, and other anthropogenic activities) impact planetary boundaries related to pollution, climate change, biodiversity, water security, food security, and other ecosystem transformations associated with health outcomes.

### Governance for health

In the absence of global government, increased attention is being given to global governance: the intergovernmental and multi-stakeholder institutions engaged in setting policies and promoting accountability and transparency at a supranational level. Some of these institutions are health specific (e.g. the World Health Organization, UNAIDS and UNICEF), others have multiple agendas (e.g. World Bank), while others have non-health agendas that nonetheless affect health outcomes within and between countries (e.g. the World Trade Organization, International Labour Organization, International Monetary Fund, and United Nations Development Program, to name a few). Several have treaty-making authority with direct or indirect global health implications. Meanwhile, the re-emergence of powerful global philanthropies and the rise of global public/private partnerships pose particular governance challenges, while the proliferation of global commissions (as knowledge generators capable of influencing norms, but not actual decision-making) presents both opportunity (continuing to refine what a global government playbook might look like) and threat (creating a lot of conceptual noise that could obfuscate underlying issues of power imbalances and wealth inequalities). Papers submitted under this topic examine global governance opportunities and risks, including studies of power politics, conflicts of interest, membership and membership criteria, effectiveness in creating health equitable policies and programs at international and national scales, critical debates over the regulatory regimes or framework conventions versus voluntary corporate social responsibility initiatives, the health impacts of international health treaties and human rights covenants, and the role of national or global taxation policies or agreements in promoting health equity within and between countries.

### Health in foreign policy

Related to issues of global governance for health and globalization and health development, a new area of research and policy scholarship examines how health becomes identified as a priority issue within country’s foreign policy deliberations, how this in turns affects debates and decisions at a global scale, and how global agreements (such as norms, declarations, and conventions) affect a country’s domestic policy environment and decision-making. At its core is how intergovernmental negotiations shape international and national decision making on health issues, how formal and informal health diplomacy is carried out and by whom, and how such actions have demonstrable impacts. Papers submitted under this topic will explore governments’ health and foreign policy positions, processes of intergovernmental negotiations, coherence (or incoherence) between differing foreign policy goals, and how different global health actors work to place health higher as both a domestic and foreign policy priority.

### Health systems

Health systems are typically thought of as the leadership, information systems, financing, human resources, products and services needed to ensure healthcare provision within a country. At a global level, international donors, grants and conditional loans have influenced the shape of these systems, affecting equity in access to care. There is a renewed global push for universal health coverage but lack of agreement on how it should be funded or administered, and how such coverage relates to older, but continually renewed, efforts to strengthen primary health care. Private financing for health systems in many countries is rising; there is a critical shortage of human resources for health; out-of-pocket payments continue to push people into ‘medical poverty’; and the high costs of new medical technologies or therapeutics too often available only to the few challenge appropriate funding levels for comprehensive primary health services essential to the many. Health care in conflict situations post particularly complex challenges for effective global response. Papers submitted under this topic explore how globalization-related processes are affecting the development of national and regional health systems, with a focus on how such systems improve health equity in terms of access, coverage, and financing.

### Migration and mobilities

Migration, the movement of people across political jurisdictions, has long been an axiomatic element of globalization, both old and new. The increased flows of refugees and internally displaced populations today, however, rank amongst the most critical political issues facing nations and international governing institutions. With population densities and resource demands increasing, and with larger numbers of the ‘Global South’ seeking access to the ‘Global North’, xenophobic sentiments are stirred, with increases in gendered, ethnic and religious discrimination. Governmental, intergovernmental and international humanitarian efforts struggle to find ways to intervene to protect the health of affected populations. At the same time as borders are increasingly closed to some migrants and refugees, they are increasingly open to ‘economic’ migrants and highly skilled individuals, including health workers. The flows of such individuals from poorer to richer countries has been argued as exacerbating global health inequities (although not all agree that it does), even as patients with the financial means are able to cross borders to seek medical care, posing both risks and benefits to both home and destination countries. Papers submitted under this topic will focus on all forms of international mobilities, and the role played by globalization processes in their dynamics, and in how they increase or reduce inequities in global health.

### Psychosocial impacts

Cutting across many of the Sustainable Development Goals, mental health is an inextricable component of physical health and general wellbeing, and an important component to achieving health for all. Psychological, neurological, and substance-misuse-related disorders contribute substantially to the global burden of disease. Biological, cultural and socio-economic factors, including living through armed conflicts, play an important role in the emergence of psychological distress, and in the ways in which they are subsequently treated. The rise of globalization, and its association with increased urbanisation, shifting individual and collective identities, and economic inequities, has not only resulted in changes in the way that mental health is identified and treated globally, but has also resulted in a shift from more indigenous expressions of mental distress towards expressions more in line with ‘Western’ nosologies. Papers submitted under this topic will explore how globalization processes are affecting mental health outcomes (from psychosocial distress to severe psychiatric disorders), and how national and regional experiences in prevention and mitigation might be scaled up internationally.

### Theory, models and methods

As a journal on a cutting edge of globalization and health research and scholarship, we encourage papers that develop critical insights into theoretical conceptualizations, explanatory models, analytical frameworks, and new methodologies that increase the evidence base for informed national and international global health decision-making.

## Moving forward

Finally, the editorial process for the journal has also been revamped, with the appointment of a larger number of Deputy Editors with particular remits for each of these Sections. There is a new Managing Editor, Dr. Corinne Packer (who works closely with me at the University of Ottawa), and new manuscript managing processes are being implemented with the support of the publisher, BMC. The intent of these changes is twofold: First, we hope to accelerate the process between submission, peer review, final journal decision, and, if accepted, production and publication. Second, we will be better able to ensure that the journal remains in the forefront of disseminating research findings and policy analyses that can meet the challenge issued in 2002 by the Commission on the Social Dimension of Globalization, if anything more relevant today than then:

The current path of globalization must change. Too few share in its benefits. Too many have no voice in its design and no influence in its course [9].
